# Trajectory of blood pressure change during pregnancy and the role of pre-gravid blood pressure: a functional data analysis approach

**DOI:** 10.1038/s41598-017-06606-0

**Published:** 2017-07-24

**Authors:** Minxue Shen, Hongzhuan Tan, Shujin Zhou, Graeme N. Smith, Mark C. Walker, Shi Wu Wen

**Affiliations:** 1 Department of Dermatology, Xiangya Hospital, Central South University, Changsha, Hunan China; 2 Department of Epidemiology and Health Statistics, Xiangya School of Public Health, Central South University, Changsha, Hunan China; 3 OMNI Research Group, Department of Obstetrics and Gynecology, Faculty of Medicine, University of Ottawa, Ottawa, Ontario Canada; 4 Ottawa Hospital Research Institute, Clinical Epidemiology Program, Ottawa, Ontario Canada; 5 Department of Maternal and Child Health Care, Liuyang Maternal and Child Hospital, Liuyang, Hunan China; 6 Department of Obstetrics and Gynecology, Queen’s University, Kingston, Ontario Canada; 7 School of Epidemiology, Public Health, and Preventive Medicine, Faculty of Medicine, University of Ottawa, Ottawa, Ontario Canada

## Abstract

The study aims to examine the blood pressure (BP) trajectory during pregnancy and its association with pre-gravid BP level. In a pre-conception cohort study, newly-married women in Liuyang, China underwent pre-gravid measurements and were followed throughout the pregnancy. BP was measured at pre-conception and again throughout pregnancy. The functional principal component analysis was used to examine the trajectory of BP changes during pregnancy. A total of 1282 women with a singleton pregnancy who had both pre-conception and gestational BP measurements performed were included in the final analysis. The results showed that BP decreased significantly in early pregnancy and increased thereafter, without BP drop around 20 weeks of gestation. Pre-gravid BP level was inversely associated with the BP drop in early pregnancy, such that women with higher pre-gravid BP had greater BP drop at the beginning, while women with the lowest pre-gravid BP level demonstrated no obvious BP drop throughout the entire pregnancy.

## Introduction

The maternal cardiovascular and metabolic systems undergo significant adaptations during pregnancy, sustaining sufficient tissue oxygenation and nutrient supply for the mother and developing fetus^1^. Previous studies have reported changes in blood pressure (BP) throughout pregnancy, and it was generally accepted that in clinically healthy pregnant women, BP falls gradually at first trimester, reaching a nadir around 22–24 weeks, rising again from 28 weeks, and reaching preconception levels by 36 weeks of gestation^2–6^. This concept has remained largely unchallenged, probably because of the well-established physiologic background; the second wave of trophoblastic invasion creates a low-resistance vascular blood flow, with the consequence of a reduced BP at around 20 weeks of gestation^7, 8^.

However, this general trend of BP variation during pregnancy has been challenged in a prospective study in homogenous population: BP increased progressively throughout the pregnancy without mid-trimester drop^9^. A few studies also reported the absence of a mid-trimester BP drop during pregnancy^10–14^. The pattern of BP changes during pregnancy has not yet been examined in Asian populations. Moreover, most of the studies had small sample sizes without pre-conception BP measurement. Neglecting the pre-gravid BP level and using average BP values may mask the variations that happen on an individual level.

Time series data are commonly treated as multivariate data because they are given as a finite discrete time series^15^. However, methods dealing with longitudinal data like local regression and generalized additive model suffer from issues associated with the dependent nature of repeated measurements within each subject. Moreover, sparse data are quit common in clinical practice, but subject with different number of measurement will be weighted differently in such models. Functional data analysis (FDA) is an approach towards modeling time series data that has started to receive attention in the literature^16^. FDA essentially treats the whole curve (obtained from a subject) as a single entity, and it is not necessarily based on the assumption that the values observed at different times for a single subject are independent. The most popular technique in FDA is functional principal component analysis (FPCA), an important dimension reduction tool. In sparse data situations, it can be used to impute functional data that are sparsely observed^17^.

We therefore conducted a prospective study of a cohort of pre-pregnant and subsequently pregnant women in China, based on the FPCA method, in order to determine the pattern of BP change in pregnancy as well as its association with pre-gravid BP level.

## Materials and Methods

### Study design

This is an ongoing prospective cohort of women in Liuyang county of Hunan province in China^18, 19^. Participants who planned to have a baby within six months were being recruited from Liuyang Maternal and Infant Hospital (where the family planning referral center and marriage registration office are located) at the time of marriage registration or pre-marriage medical examination. Women were assessed at recruitment, and followed across the pregnancy up to delivery when they subsequently become pregnant.

### Blood pressure measurements

Systolic blood pressure (SBP) and diastolic blood pressure (DBP) were measured by clinical staff at recruitment using automated non-invasive blood pressure monitors BpTRU, after the woman had rested for 10 min and were comfortably seated with the back supported. The left upper arm was at heart level and supported, and an appropriate cuff was selected for the woman. BpTRU is an automated oscillometric non-invasive BP monitor that is reliable and reduces the “white-coat” effect. Automatic mode was selected to obtain a series of six BP measurements, with a 2-minute interval between measurements. The clinical staff left the woman alone in the room after the first measurement. The first BP measurement was discarded, and the average BP value of the rest five measurements was obtained and recorded. Mean arterial pressure (MAP) was calculated as DBP + (SBP − DBP)/3 (mmHg).

### Baseline assessment

All participating women underwent a baseline pre-gravid assessment which includes: (1) interviewer-administered questionnaire (demographic characteristics and medical history); (2) physical examination (measurement of height, weight, and BP). Body mass index (BMI) was calculated as weight [kg]/(height [m])^2^.

### Statistical analyses

Antenatal BP measurements were sparsely distributed along the timeline of pregnancy, ranging from 5 to 41 weeks of gestation. BP change (%) in pregnancy was defined as: [(BP in pregnancy − pre-gravid BP)/pre-gravid BP] × 100%. Normotensive women with complete baseline information and three or more antenatal visits were included in the analysis. Women with only one or two antenatal visits were excluded (N = 28) in order to obtain more stable estimates.

Characteristics of the participants were described using means and standard deviation for continuous variables, and using percentage for categorical variables. Parity was categorized as nulliparous and parous. Characteristics of the included and excluded women were compared using Student *t* test or chi-square test.

FPCA for sparse longitudinal data was applied to obtain the trajectories of BP change during pregnancy. First, the cubic B-spline basis function was chosen for representing the eigenfunctions. The restricted maximum likelihood estimation through a Newton-Raphson procedure was used to estimate functional components from sparse longitudinal data. Number of basis functions and nonzero eigenvalues were determined by minimizing an approximation of the leave-one-curve-out cross-validation score. Stratification by pre-gravid MAP tertiles was used to examine the phenotype of longitudinal BP changes in pregnancy.

Subgroup analyses were performed between women having less than six visits (the median) and those having six or more visits. FPCA was performed in R Statistical Software using the package “fpca”. Data cleaning and other statistical analyses were performed in SAS version 9.4 (SAS Institute Inc., Cary, North Carolina, USA). The significance level was 0.05 for all statistical tests.

### Ethics statement

This study was conducted according to the guidelines laid down in the Declaration of Helsinki. All procedures involving patients were approved by the institutional research ethics boards of Central South University (Changsha, China), Ottawa Hospital Research Institute (Ottawa, Canada) and Mount Sinai Hospital (Toronto, Canada). Written informed consent was obtained from all women.

## Results

**Figure 1:**
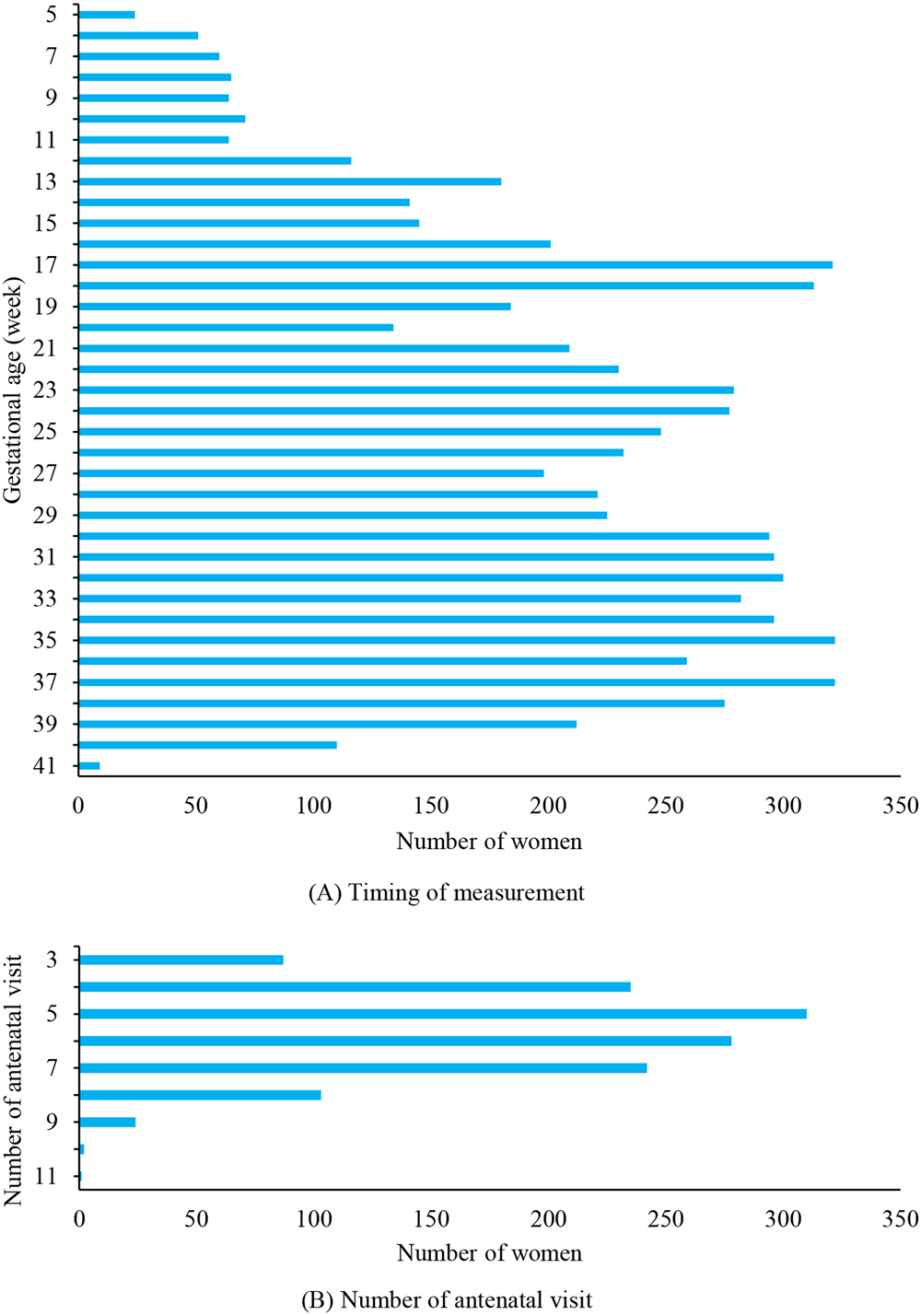
Distribution of the timing of measurements and number of antenatal visits.

3375 women have been recruited to our cohort from April 2009 to February 2012. A total of 2292 women have completed a pregnancy and had a singleton delivery; 1482 had antenatal check-up data; 1282 normotensive women with complete baseline information and three more antenatal BP measurements were included in the final analysis. The distribution of the timing of measurements and number of antenatal visits are shown in Fig. 1. A majority of women had four to seven antenatal visits.

The median time between baseline assessment and singleton pregnancy was 26.3 weeks. Baseline characteristics of the study participants are shown in Table 1. The 1282 women included in the final analysis did not differ from the rest of the women with singleton birth (n = 1010) except years of education, although the difference was subtle.

**Table 1:** Comparison of characteristics of included and not included participants.

	Women included in analysis	Women excluded from analysis	*P*
N	1282	1010	
Age (years)	25.3 ± 3.2	25.3 ± 2.9	0.89
Education (years)	10.8 ± 2.3	11.4 ± 2.7	<0.01
Pre-gravid BMI (kg/m^2^)	20.2 ± 2.3	20.0 ± 2.6	0.17
Pre-gravid SBP (mmHg)	110.9 ± 12.0	111.6 ± 11.7	0.13
Pre-gravid DBP (mmHg)	71.0 ± 8.9	70.9 ± 8.7	0.80
Parity			
Nulliparous	1111 (86.7%)	884 (87.5%)	0.57
Parous	171 (13.3%)	126 (12.5%)	

**Figure 2:**
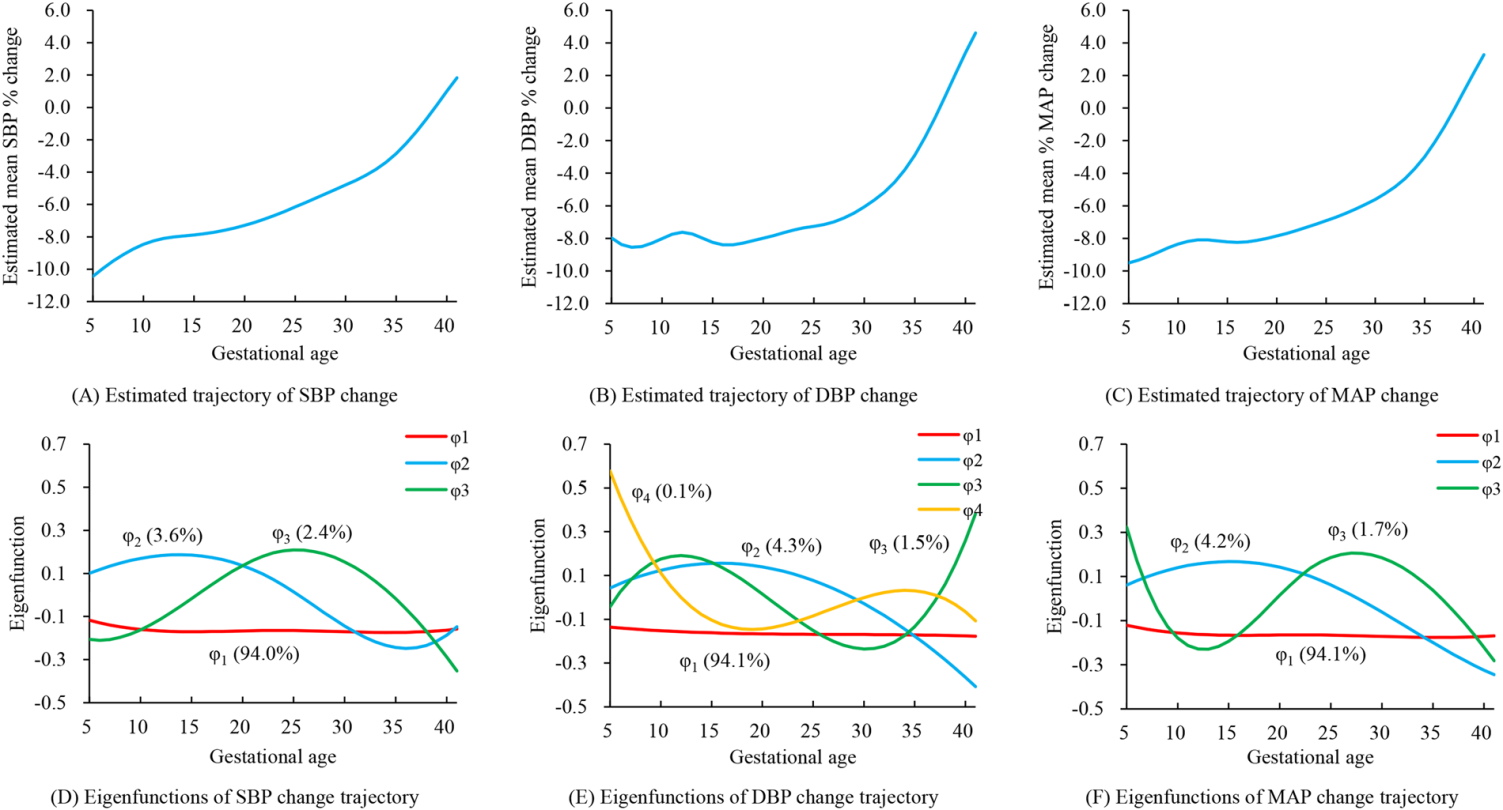
Estimated trajectories of blood pressure change from pre-gravid level and the eigenfunctions. Top panels showed the trajectories of per cent blood pressure change from the pre-gravid level, estimated by functional principal component analysis. Bottom panels showed the first three or four eigenfunctions. Order of eigenfunctions and the percentage of the variation explained by each eigenfunction were expressed as φ_i_ (%).

The estimated trajectories of SBP, DBP and MAP are shown in top panels of Fig. 2. Three to four eigenfunctions were selected by FPCA, as shown in bottom panels of Fig. 2. The first eigenfunction explained most of the total variation (>90%). Overall, BP dropped at the very beginning of pregnancy as compared to the pre-gravid BP, and then increased slowly throughout first and second trimester, and finally resumed the pre-gravid level in late pregnancy. No obvious BP drop was observed around 20 weeks of gestation, although some fluctuations around 12 weeks were present.

**Figure 3:**
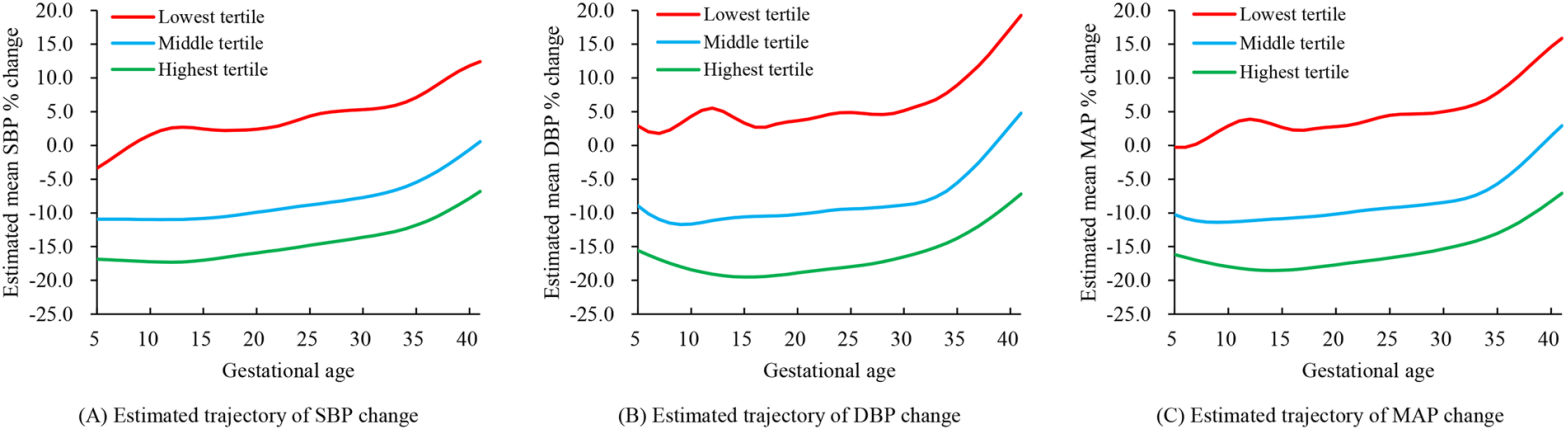
Estimated trajectories of blood pressure change from pre-gravid level, stratified by the pre-gravid mean arterial pressure tertile. Stratifications were created according to the pre-gravid mean arterial pressure tertiles. Different colors signified different tertiles. Women in the highest pre-gravid MAP tertile had the largest BP drop at the beginning of pregnancy, while women in the lowest MAP tertile showed no BP drop at the beginning. Women in the highest tertile showed DBP drop around 15 weeks of gestation. Women in the lowest tertile showed some BP fluctuations at the before 15 weeks of gestation.

In order to investigate how pre-gravid BP level impact BP changes during pregnancy, stratification analysis was performed according to pre-gravid MAP tertiles (Fig. 3). Phenotypes of trajectories were identified: women in the highest pre-gravid MAP tertile had the largest BP drop at the beginning of pregnancy; by contrast, those in the lowest tertile did not present significant BP drop throughout the pregnancy. Interestingly, women in the highest tertile presented subtle DBP drop around 15 weeks of gestation (Fig. 3B).

**Figure 4:**
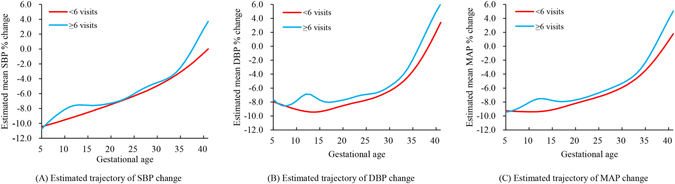
Estimated trajectories of blood pressure change from pre-gravid level, stratified by the number of antenatal visit. The sample was divided by the median of antenatal visits. Subgroup analysis showed that both subgroups had similar trajectories and trends, partly overlapped, although fluctuations were present. No major selection bias existed.

Subgroups analysis compared the trajectories estimated from women having less than six visits and those having six or more. As shown in Fig. 4, both subgroups had similar trajectories and dynamic trends, partly overlapped, although fluctuations were present between 10 and 15 weeks of gestation. Major selection bias was not likely to occur according to the subgroup analysis.

## Discussion

The study applied functional data analysis for the spare longitudinal antenatal BP measurements, and showed that BP dropped at the beginning of pregnancy as compared to pre-gravid BP level, and increased throughout the pregnancy, with no significant BP drop around 20 weeks of gestation. Pre-gravid BP level was associated with the phenotypes of gestational BP trajectories. It was inversely associated with the BP drop in early pregnancy, such that women with higher pre-gravid BP level presented greater BP drop from the beginning, while women with the lowest pre-gravid BP level demonstrated no obvious BP drop throughout the entire pregnancy. Our data also contradicted the current dogma that there is a mid-trimester drop in BP, addressed the gap in the literature with respect to the absence of pre-pregnancy measurements.

The majority of previous studies did not measure pre-pregnancy BP, and reports on the pattern of BP changes were usually limited within the pregnancy. Studies reporting a mid-trimester BP drop were heterogeneous with respect to measurement device, time of measurement, study design, inclusion criteria, sample size, and statistical methods. Some of the previous observations were descriptive and statistical approaches were outdated. Repeated measurement nested within individuals are highly dependent on each other, and neglect of this intra-class correlation by treating each measurement as an independent observation will lead to erroneous statistical conclusions^20^. Our finding corroborates the work by Nama *et al*. that reported a progressive increases in mean SBP and DBP throughout healthy pregnancy without mid-trimester BP drop^9^. In that study, participants were homogenous population (white normotensive and primiparous women), and statistical methods were robust in dealing with intra-class correlation. Their finding undermines the widely accepted mid-trimester drop in BP. However, the homogeneity of study population limited the generalization of their conclusion. Moreover, the timing of measurement was categorized into several intervals in that study and most of the related studies owing to the deficiency of models to deal with sparse longitudinal data. In our study, participants were healthy Chinese women who remained normotensive throughout the pregnancy. The included 1310 women did not differ from the excluded participants with singleton birth with respect to most of the baseline characteristics. We applied the FDA method to obtain more stable and robust estimates for trajectories. The method treats the whole curve as a single entity, and there is no concern about the intra-individual correlations.

Several limitations of our study warrant discussion. First, as participants were recruited before conception, the time interval between recruitment and pregnancy was uncontrollable, although it is unlikely that the interval would not have influence on the trends of serial measurements of BP during pregnancy. Second, since the study population consisted of young recently-married Chinese women, our findings may limit its generalizability to non-Chinese populations. Last, selection bias might exist in our study because we selected a subgroup of participants who had visits during pregnancy than had the rest of the cohort, although no differences were found with respect to major baseline characteristics between included and excluded women, and sensitivity analysis demonstrated consistent results among women having different number of antenatal visits.

Despite the limitations, our study has a number of strengths. First, the sample size of our study is relatively large, which ensures enough power to examine even small differences. Second, we used FDA method to estimate the trajectories of antenatal BP change. The method could perfectly deal with sparse longitudinal data with intra-individual correlations. Third, we have been able to measure pre-conception BP so that we could examine the pattern of BP from preconception to pregnancy and pre-gravid determinants of BP variations in a prospective way, resulting in novel insights that should lead to further studies.

The mechanism why pre-gravid BP was inversely associated with the BP drop at the beginning of pregnancy is not clear. To our knowledge, this is the first study that reported the phenotypes of BP trajectories associated with the pre-gravid BP level. The magnitude of this association was surprisingly high. We speculated that the phenomenon might be associated with a BP homeostasis mechanism, i.e., women with low pre-gravid BP level had no obvious BP drop during pregnancy in order to sustain sufficient oxygen and nutrient supply for the developing fetus. Nevertheless, further studies are needed to validate the phenomenon and to discuss its implications for clinical practice.
